# How to Choose In Vitro Systems to Predict In Vivo Drug Clearance: A System Pharmacology Perspective

**DOI:** 10.1155/2015/857327

**Published:** 2015-10-11

**Authors:** Lei Wang, ChienWei Chiang, Hong Liang, Hengyi Wu, Weixing Feng, Sara K. Quinney, Jin Li, Lang Li

**Affiliations:** ^1^Bioinformatics Research Center, College of Automation, Harbin Engineering University, Harbin, Heilongjiang 150001, China; ^2^Biomedical Engineering Institute, College of Automation, Harbin Engineering University, Harbin, Heilongjiang 150001, China; ^3^Center for Computational Biology and Bioinformatics, School of Medicine, Indiana University, Indianapolis, IN 46202, USA; ^4^School of Informatics and Computing, Indiana University, Indianapolis, IN 46202, USA; ^5^Pattern Recognition and Intelligent System Institute, College of Automation, Harbin Engineering University, Harbin, Heilongjiang 150001, China; ^6^Department of Obstetrics and Gynecology, School of Medicine, Indiana University, Indianapolis, IN 46202, USA; ^7^Department of Medical and Molecular Genomics, School of Medicine, Indiana University, Indianapolis, IN 46202, USA

## Abstract

The use of in vitro metabolism data to predict human clearance has become more significant in the current prediction of large scale drug clearance for all the drugs. The relevant information (in vitro metabolism data and in vivo human clearance values) of thirty-five drugs that satisfied the entry criteria of probe drugs was collated from the literature. Then the performance of different in vitro systems including *Escherichia coli* system, yeast system, lymphoblastoid system and baculovirus system is compared after in vitro-in vivo extrapolation. Baculovirus system, which can provide most of the data, has almost equal accuracy as the other systems in predicting clearance. And in most cases, baculovirus system has the smaller CV in scaling factors. Therefore, the baculovirus system can be recognized as the suitable system for the large scale drug clearance prediction.

## 1. Introduction

In vivo drug clearance is a very significant pharmacokinetic parameter, which largely determines the drug exposure in human body [1, 2]. Predicting the clinical in vivo drug clearance from the preclinical in vitro experiments is essential during the drug development. Specifically, hepatic clearance (CL_H_) is the most important clearance parameter as the majority of the drugs are metabolized in human liver [3].

The most common in vitro drug clearance methods include the use of human liver microsomes (HLMs) or hepatocytes [4], which are well documented in the literature [5–8]. The advantage of HLMs and human hepatocytes is that they are physiologically closer to human liver [4, 9, 10]. Their disadvantages include enormous problems between sample variations with unknown causes and relative high expense [11, 12]. In particular, the large variation of in vitro experiments in HLMs or hepatocytes causes the doubts in reproducibility. On the other hand, the commercial availability of recombinant human enzyme expression systems makes the prediction of human drug clearance cheaper and more reproducible [13, 14]. The advantages and limitations of each in vitro approach are well documented [15–21].

In order to predict in vivo clearance from in vitro experiments, system pharmacology model, such as the physiologically based pharmacokinetic (PBPK) model have been developed rapidly [22–25]. Yap et al. [26] present statistical learning models based on mixed physicochemical and topological descriptors. Demir-Kavuk et al. [27] develop a single application called DemQSAR. Simcyp [28] and Gastroplus [29] are developed originally in collaboration with major pharmaceutical companies to simulate and predict drug clearance and drug interaction in virtual patient populations.

Despite the previously described advances in both technology and system pharmacology modeling, in vitro drug clearance prediction still faces some new challenges [25, 30]. There are a number of in vitro recombinant enzyme systems available, but it is not clear whether they all perform similarly or differently. The performance of different recombinant systems can also be enzyme dependent, but little was known about it [30]. Finally and most importantly, unlike traditional physiologically based pharmacokinetics modeling that investigates one or a few drugs a time, current translational bioinformatics desires a system model that can conduct large scale drug clearance for all the drugs [31]. This is a new challenge that poses not only the accuracy of the in vitro-in vivo clearance prediction, but also the completeness and variations of the annotated in vitro recombinant experiment data on drug metabolisms. As the in vitro-in vivo clearance prediction has been well documented in the literature, this paper will address the completeness and variations of various in vitro recombinant experiments.

## 2. Methods

### 2.1. In Vitro Experimental Data Collection

All the in vitro data of selected drugs were collated from the published literature after identifying sources using PubMed. The criteria for drug selection were that they were recognized as probes for specific cytochrome P450s (CYP) or metabolized mostly by a single CYP enzyme [49, 50]. Probe drugs refer to drugs whose plasma AUC values had been shown to increase 5-fold or higher when coadministered with a known CYP inhibitor or AUC ratio in poor metabolizers versus 1280 extensive metabolizers is greater than 5-fold [50]. These literature data including *V*_max⁡_ (pmol min^−1 ^pmol^−1^CYP), *K*_*m*_ (pM) were obtained from various systems for heterologous expression of recombinant P450 enzymes containing bacterial expression in* Escherichia coli*, expression in yeast cells, lymphoblastoid expression systems, and baculovirus-driven expression in insect cells. Fraction unbound in plasma (fu) of drugs was also collected. If intervals of the fraction of drug unbound in plasma parameters were collected, the mean of an interval was the acceptable value.

### 2.2. In Vivo Data Collection

Human clearance values were taken from published original work and in part reported by Obach et al. [32]. Both intravenous data and oral data were accepted. In the case of oral clearance, the clearance was taken as a product of oral clearance and absolute bioavailability of the drug, in order to calculate drugs' intravenous clearance. The bioavailability was got through Drug Bank [39] and published original literature with a single point or the mean of an interval. At the end, only the intravenous clearance was used to assess in vitro-in vivo clearance prediction.

### 2.3. In Vitro-In Vivo Extrapolation

Prediction of drug hepatic clearance using in vitro recombinant P450 enzyme kinetic parameters was performed in three main steps. Initially, intrinsic clearance per unit enzyme (CL_int,rec_) was calculated by the following:

(1) $$\begin{array}{c} \text{C}{\text{L}}_{\mathrm{int},\text{rec}}=\frac{v_{\max}}{k_{m}}. \end{array}$$

The median CL_int,rec_ value of the same recombinant P450 enzymes expression systems for each drug was taken, respectively.

After that, CL_int,rec_ was converted to a whole organ intrinsic metabolic clearance (CL_int_) using enzyme abundance, MPPGL, and the liver weight as shown in the following:

(2) CLint=∑j=1mCLint,rec·enzyme  abundance ·MPPGL·liver  weight,

where there were *m* CYPs with corresponding CL_int,rec_ values for different pathways in each recombinant system; enzyme abundance refers to the amount (pmol P450) per milligram of microsomal protein; MPPGL means the amount (mg) of microsomal protein per gram of liver; and the liver weight means the weight (g) of human liver. Enzyme abundance, MPPGL, liver weight, and liver blood flow were generated by Simcyp with 1000 Sim-healthy volunteers (age: 20–50), female/male ratio 1, and 100% of extensive metabolizer for all major CYP enzymes [51].

At last, the value of CL_int_ was combined with binding parameters (*f*_ub_) and liver blood flow (*Q*_H_) to extrapolate to whole organ clearance by well-stirred model by the following:

(3) $$\begin{array}{c} \text{C}{\text{L}}_{\text{H}}=\frac{\text{C}{\text{L}}_{\mathrm{int}}\times Q_{\text{H}}\times f_{\text{ub}}}{\text{C}{\text{L}}_{\mathrm{int}}+Q_{\text{H}}\times f_{\text{ub}}}, \end{array}$$

where *f*_ub_ is the fraction of drug unbound in blood. So, it could be calculated by *f*_u_/*B*/*P* ratios. While *B*/*P* ratios were not all available from the literature, a default value of 0.55 was used. Meanwhile, nonspecific microsomal binding was ignored.

### 2.4. Scaling Factor

The scaling factor of each probe drug was assessed from the difference between predicted and observed in vivo values as described in the following:

(4) $$\begin{array}{c} \text{Scaling}\;\;\text{Factor}=\log_{2}\left(\frac{\text{C}{\text{L}}_{\text{H},\text{in}\;\text{vivo}}}{\text{C}{\text{L}}_{\text{H},\text{predicted}}}\right), \end{array}$$

where CL_H,in vivo_ is the observed in vivo clearance and CL_H,predicted_ is the predicted value. Then, the scaling factor for different enzymes was determined by averaging scaling factor of probe drugs with the same recombinant P450 enzymes expression systems. This value also could assess the accuracy of clearance predicting. For one drug, if the scaling factor in one system was identical to the others, they had the same accuracy in predicting.

### 2.5. Statistical Analysis

All data were presented as mean ± S.E., unless stated otherwise. To measure the variability of prediction, the coefficient of variation (CV) was utilized. This CV measures the technical variations of in vitro metabolism experiments published from different labs.

## 3. Results

### 3.1. Literature Data Collection

Thirty-five drugs were considered as probe drugs for various enzymes, CYP1A2, CYP2B6, CYP2C9, CYP2C19, CYP2D6, and CYP3A, from different expression systems as they had relatively adequate kinetic data, as shown in Table 1 [49, 50].

### 3.2. Comparison of Clearance Predictions for Different Enzyme Probe Drugs from the Same Expression System

**Figure 1:**
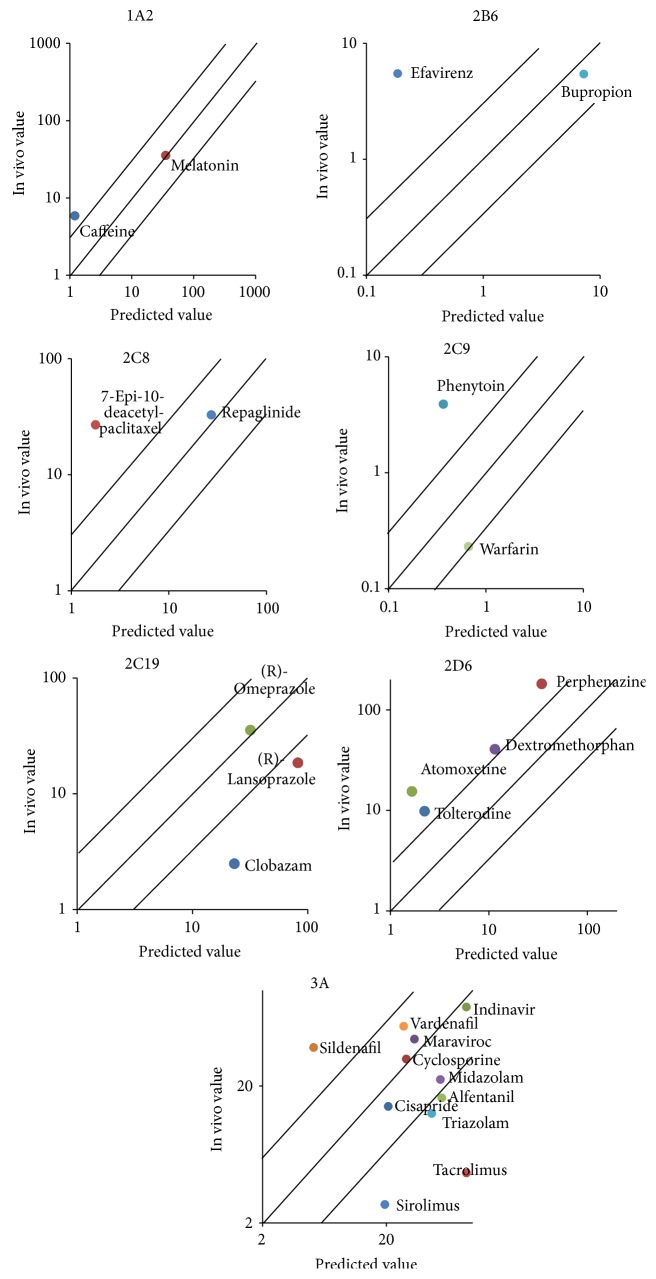
Predicted versus observed clearances of 28 drugs of baculovirus expression system.

Since most drugs had baculovirus system data, they were used to predict probe drugs' clearance. The predicted clearance was within 3-fold of the observed in vivo value for 6 of the 15 (40%) drugs for CYP3A probe drugs. While for CYP 2D6, none of the predicted values was within 3-fold the observed in vivo value. Only one drug was within 3-fold the observed value for CYP1A2, 2B6, 2C8, 2C9, and 2C19, which accounted for 50%, 50%, 50%, 50%, and 33% of the total. These results were illustrated in Figure 1 and Table 2.

### 3.3. Comparisons of Clearance Predictions in the Different Expression Systems

**Figure 2:**
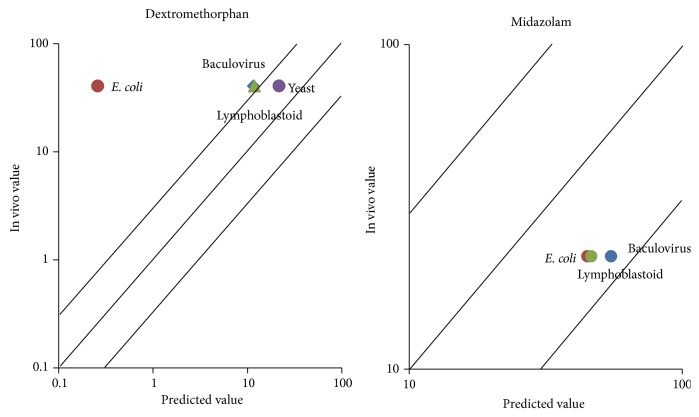
Predicted versus observed clearances of 2 drugs with different expression systems.

Dextromethorphan and midazolam were selected to compare different expression systems, because these two drugs were investigated and published under all these systems. For dextromethorphan, the predicted values from yeast system were only within 3-fold the in vivo value. And baculovirus system and lymphoblastoid system had almost the same prediction accuracy (Figure 2, Table 2).

For midazolam, all of the predicted clearance values were within 3-fold the in vivo clearance values. The most accurate predicted value was from* E. coli* system. And the three expression systems had almost the same prediction accuracy (Figure 2, Table 2).

### 3.4. Comparison of Data Availability from Different Expression Systems

**Figure 3:**
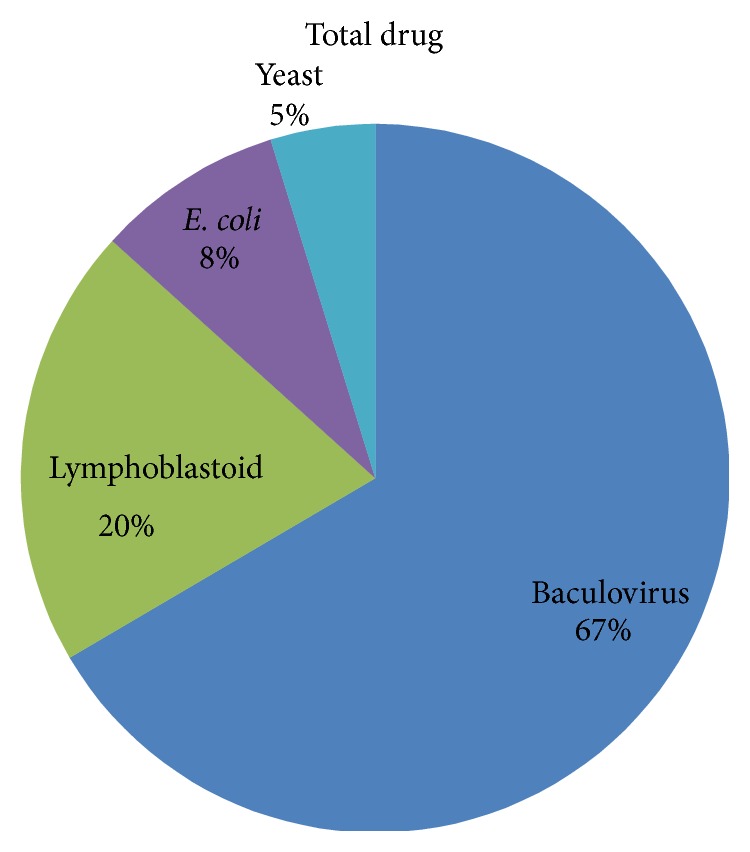
Proportion of data from different expression systems.

All the in vitro recombinant enzyme expression system data were collated from the published literature. The total number of data points was 293.  Figure 3 showed the proportion of data from different expression systems. In general, baculovirus and lymphoblastoid system were more abundant than the others. Baculovirus system has the largest proportion, 67%. Lymphoblastoid system was the second one, 20%. Only 8% and 5% of the data came from* E. coli* system and yeast system.

If we mapped all the data to different drugs, the majority of the drugs (28/35) were tested in the baculovirus expression system; part (12/35) of the selected drugs were test in the lymphoblastoid expression systems and only 4/35 were from* E. coli *and yeast systems, respectively.

### 3.5. Comparisons of Scaling Factors

**Figure 4:**
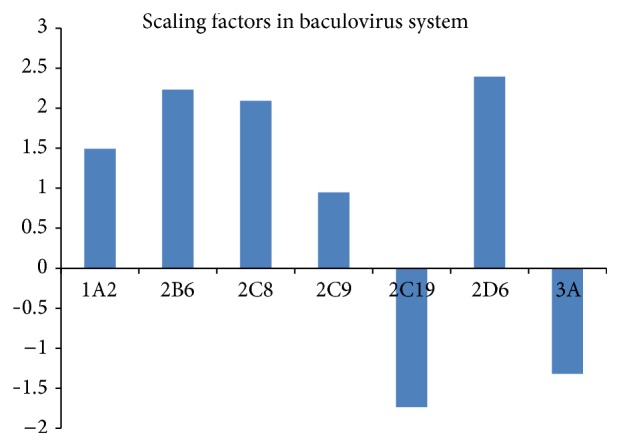
Comparisons of scaling factors in the baculovirus system.

Scaling factors of different enzymes based on CYP expression systems were calculated and shown in Table 3. These scaling factor ranged from −1.735 to 3.794. In the baculovirus expression system, the values of scaling factors varied a lot across the enzymes (Figure 4). And 71.4% (5/7) of the values, whose range was −1.735 to 2.394, were positive.

The variability in the same enzyme between CYP expression systems was also different. In CYP2D6, yeast system and lymphoblastoid system had higher variability than baculovirus system with the coefficient of variation (CV) 65.13%, 87.95%, and 25.10%, respectively. In CYP3A, lymphoblastoid system (CV = 356.60%) had higher variability than baculovirus system (CV = 200.98%) similarly. However, in CYP2C9 the coefficient of variation in lymphoblastoid (CV = 153.66%) was smaller than baculovirus system (CV = 368.53%).

## 4. Discussion and Conclusion

In this paper, we compare the performance of different recombinant human enzyme expression systems (including* Escherichia coli* system, yeast system, lymphoblastoid system, and baculovirus system) for predicting hepatic clearance in human body. And we attempt to find out the most suitable one for the large scale drug clearance prediction. After collecting the in vitro pharmacokinetic parameters of thirty-five probe drugs, we use in vitro-in vivo extrapolation to predict the clearance. The experimental results (Table 2) show that half (24/48) of the predicted values in different in vitro systems are within 3-fold the observed in vivo clearance values.

The comparisons of clearance predictions for different enzyme probe drugs from the same expression system and in different expression systems, data availability from different expression systems, and scaling factors are further analyzed. Figure 2 shows that baculovirus system has almost equal accuracy as the other systems in predicting clearance. Meanwhile, it can provide more and sufficient data for prediction than the others (Figure 3). We should note that the scaling factor will be enzyme dependent as shown in Table 3 and in most cases baculovirus systems have the smaller CV in scaling factors. Therefore, we shall use data of the baculovirus system for the large scale drug clearance prediction.

Nevertheless, there are a few more caveats. Most important of all is that in vivo clearance of some probe drugs we collected contains the renal clearance. Some of the in vivo clearance is obtained as the systemic clearance. And the proportion of hepatic metabolism was not clear. Hence, the scaling factor estimation may have some bias.

In most closely related studies, the combination of HLM and recombinant enzymes is implemented to predict in vivo clearance for high accuracy of in vitro-in vivo extrapolation [52–54]. But most of them only focused on one drug, and the choice of in vitro systems was not taken into consideration.

We are fully aware that some drugs are metabolized through non-CYP pathways, such as oxidases, reductases, and other phase II metabolism enzymes. Our preliminary research on these enzymes revealed very limited in vitro experiment data on only a handful of drugs. Therefore, these data cannot be scaled up to do large scale in vitro-in vivo prediction and to evaluate their variations.

To our knowledge, this is the first study to compare the performance of different in vitro systems and make a decision. With the assistance of our work, the large scale drug clearance prediction should be more effective and efficient.

## Figures and Tables

**Table 1:** Drug set.

Drug	Expression systems	In vivo clearance (L/h)	References
Caffeine	Baculovirus	5.88	Obach et al. (2008) [32]
Melatonin	Baculovirus	57.96	Mallo et al. (1990) [33]
Tacrine	Yeast	235.2	Obach et al. (2008) [32]
Theophylline (1,3-DMX)	*E. coli* and lymphoblastoid	3.612	Obach et al. (2008) [32]
Bupropion	Baculovirus	5.415	Lei et al. (2010) [34], Hill et al. (2007) [35]
Efavirenz	Baculovirus	5.483	Gengiah et al. (2012) [36], Chiappetta et al. (2010) [37]
Repaglinide	Baculovirus	32.76	Obach et al. (2008) [32]
Paclitaxel	Baculovirus	26.88	Obach et al. (2008) [32]
(R)-Warfarin	Baculovirus, *E. coli*, and lymphoblastoid	0.231	Obach et al. (2008) [32]
Phenytoin	Baculovirus and lymphoblastoid	3.906	Hayes et al. (1975) [38]
Celecoxib	Lymphoblastoid and yeast	21.05	Drug Bank [39], Paulson et al. (2001) [40]
Clobazam	Baculovirus	2.49	Drug Bank [39]
(R)-Lansoprazole (dexlansoprazole)	Baculovirus	18.48	Obach et al. (2008) [32]
(R)-Omeprazole	Baculovirus and lymphoblastoid	35.28	Obach et al. (2008) [32]
Atomoxetine	Baculovirus	15.435	Drug Bank [39]
Dextromethorphan	Baculovirus, *E. coli*, yeast, and lymphoblastoid	40.59	Moghadamnia et al. (2003) [41], Kukanich and Papich (2004) [42]
Metoprolol	Lymphoblastoid	54.6	Obach et al. (2008) [32]
Perphenazine	Baculovirus	113.4	Obach et al. (2008) [32]
Tolterodine	Baculovirus	10.5	Brynne et al. (1997) [43]
Venlafaxine	Lymphoblastoid and yeast	40.95	Drug Bank [39]
Alfentanil	Baculovirus	16.38	Obach et al. (2008) [32]
Astemizole	Yeast	82.6	Lefebvre et al. (1997) [44]
Cisapride	Baculovirus	14.20	Lowry et al. (2003) [45]
Cyclosporine	Baculovirus	31.5	Obach et al. (2008) [32]
Felodipine	Baculovirus and lymphoblastoid	46.2	Obach et al. (2008) [32]
Indinavir	Baculovirus	75.6	Obach et al. (2008) [32]
Maraviroc	Baculovirus	44	Abel et al (2008) [46]
Midazolam	Baculovirus, *E. coli*, and lymphoblastoid	22.26	Obach et al. (2008) [32]
Pimozide	Baculovirus	0.042	Desta et al. (1999) [47]
Quinidine	Lymphoblastoid	16.8	Obach et al. (2008) [32]
Sildenafil	Baculovirus	38.22	Obach et al. (2008) [32]
Sirolimus	Baculovirus	2.73	Brattstram et al. (2000) [48]
Tacrolimus	Baculovirus	4.63	Obach et al. (2008) [32]
Triazolam	Baculovirus and lymphoblastoid	12.6	Obach et al. (2008) [32]
Vardenafil	Baculovirus	54.6	Obach et al. (2008) [32]

**Table 2:** Predicted value and observed in vivo value of probe drugs.

Drug	Expression systems	The predicted value (L/h)	The observed in vivo value (L/h)	Scaling factor
Caffeine	Baculovirus	1.21	5.88	2.28
(R)-Lansoprazole (dexlansoprazole)	Baculovirus	81.74	18.48	−2.12
(R)-Omeprazole	Baculovirus	31.81	35.28	0.15
(R)-Omeprazole	Lymphoblastoid	15.56	35.28	1.18
(R)-Warfarin	Baculovirus	0.66	0.231	−1.51
(R)-Warfarin	*E.coli *	0.0041	0.231	5.82
(R)-Warfarin	Lymphoblastoid	0.018	0.1512	3.07
7-Epi-10-deacetyl-paclitaxel	Baculovirus	1.77	26.88	3.93
Alfentanil	Baculovirus	56.32	16.38	−1.79
Astemizole	Yeast	40.24	82.6	1.04
Atomoxetine	Baculovirus	1.65	20.99875	3.67
Bupropion	Baculovirus	7.27	5.415	−0.43
Celecoxib	Lymphoblastoid	41.26	21.05	−0.97
Celecoxib	Yeast	5.66	21.05	1.90
Cisapride	Baculovirus	20.76	14.1975	−0.56
Clobazam	Baculovirus	23.02	2.49	−3.18
Cyclosporine	Baculovirus	29.07	31.5	0.11
Dextromethorphan	Baculovirus	11.53	15.435	0.42
Dextromethorphan	*E.coli *	0.26	15.435	5.89
Dextromethorphan	Lymphoblastoid	11.87	15.435	0.38
Dextromethorphan	Yeast	21.66195	15.435	−0.49
Efavirenz	Baculovirus	0.19	5.483	4.85
Felodipine	Lymphoblastoid	0.83	46.2	5.80
Indinavir	Baculovirus	89.11	75.6	−0.23
Maraviroc	Baculovirus	33.78	44	0.38
Melatonin	Baculovirus	35.52	57.96	0.70
Metoprolol	Lymphoblastoid	44.05	54.6	0.31
Midazolam	Baculovirus	54.9	22.26	−1.29
Midazolam	*E.coli *	44.97	22.26	−1.03
Midazolam	Lymphoblastoid	46.51	22.26	−1.06
Perphenazine	Baculovirus	34.35	113.4	1.72
Phenytoin	Baculovirus	0.37	3.906	3.40
Phenytoin	Lymphoblastoid	1.07	3.906	1.87
Pimozide	Baculovirus	6.87	0.042471	−6.64
Quinidine	Lymphoblastoid	76.93	16.8	−2.18
Repaglinide	Baculovirus	27.29	32.76	0.26
Sildenafil	Baculovirus	5.17	38.22	2.89
Sirolimus	Baculovirus	19.47	2.73	−2.84
Tacrine	Yeast	16.96	235.2	3.79
Tacrolimus	Baculovirus	89.11	4.634	−4.32
Theophylline (1,3-DMX)	*E. coli *	6.6	3.612	−0.86
Theophylline (1,3-DMX)	Lymphoblastoid	8.04	3.612	−1.15
Tolterodine	baculovirus	2.22	10.5	2.24
Triazolam	Baculovirus	46.65	12.6	−1.89
Triazolam	Lymphoblastoid	4.65	12.6	1.44
Vardenafil	Baculovirus	27.75	54.6	0.98
Venlafaxine	Lymphoblastoid	13.91	40.95	1.56
Venlafaxine	Yeast	2.76	40.95	3.89

**Table 3:** Scaling factor with different enzymes and expression systems.

Enzymes	Expression systems	Scaling factor (mean ± SD)	CV
1A2	Baculovirus	1.493 ± 1.112	74.48%
1A2	*E. coli *	−0.869	—
1A2	Lymphoblastoid	−1.154	—
1A2	Yeast	3.794	—
2B6	Baculovirus	2.232 ± 3.756	168.28%
2C8	Baculovirus	2.093 ± 2.588	123.65%
2C9	Baculovirus	0.947 ± 3.490	368.53%
2C9	*E. coli *	5.828	—
2C9	Lymphoblastoid	1.530 ± 2.351	153.66%
2C9	Yeast	1.894	—
2C19	Baculovirus	−1.735 ± 1.716	98.90%
2C19	Lymphoblastoid	1.181	—
2D6	Baculovirus	2.394 ± 0.601	25.10%
2D6	Lymphoblastoid	1.213 ± 0.790	65.13%
2D6	*E. coli *	7.290	—
2D6	Yeast	2.399 ± 2.111	87.95%
3A	Baculovirus	−1.320 ± 2.653	200.98%
3A	*E. coli *	−1.014	—
3A	Lymphoblastoid	0.993 ± 3.541	356.60%
3A	Yeast	1.038	—

## References

[1] Abduljalil K., Cain T., Humphries H., Rostami-Hodjegan A. (2014). Deciding on success criteria for predictability of pharmacokinetic parameters from in vitro studies: an analysis based on in vivo observations. *Drug Metabolism & Disposition*.

[2] Lombardo F., Obach R. S., Varma M. V., Stringer R., Berellini G. (2014). Clearance mechanism assignment and total clearance prediction in human based upon in silico models. *Journal of Medicinal Chemistry*.

[3] Shou M. (2005). Prediction of pharmacokinetics and drug-drug interactions from in vitro metabolism data. *Current Opinion in Drug Discovery and Development*.

[4] Zhang D., Luo G., Ding X., Lu C. (2012). Preclinical experimental models of drug metabolism and disposition in drug discovery and development. *Acta Pharmaceutica Sinica B*.

[5] Tang H., Mayersohn M. (2005). A novel model for prediction of human drug clearance by allometric scaling. *Drug Metabolism and Disposition*.

[6] Stringer R. A., Strain-Damerell C., Nicklin P., Houston J. B. (2009). Evaluation of recombinant cytochrome p450 enzymes as an in vitro system for metabolic clearance predictions. *Drug Metabolism & Disposition*.

[7] Zanelli U., Caradonna N. P., Hallifax D., Turlizzi E., Houston J. B. (2012). Comparison of cryopreserved HepaRG cells with cryopreserved human hepatocytes for prediction of clearance for 26 drugs. *Drug Metabolism and Disposition*.

[8] Ito K., Houston J. B. (2005). Prediction of human drug clearance from in vitro and preclinical data using physiologically based and empirical approaches. *Pharmaceutical Research*.

[9] Sahi J., Grepper S., Smith C. (2010). Hepatocytes as a tool in drug metabolism, transport and safety evaluations in drug discovery. *Current Drug Discovery Technologies*.

[10] McGinnity D. F., Soars M. G., Urbanowicz R. A., Riley R. J. (2004). Evaluation of fresh and cryopreserved hepatocytes as in vitro drug metabolism tools for the prediction of metabolic clearance. *Drug Metabolism and Disposition*.

[11] Zhao P., Kunze K. L., Lee C. A. (2005). Evaluation of time-dependent inactivation of CYP3A in cryopreserved human hepatocytes. *Drug Metabolism & Disposition*.

[12] Hariparsad N., Sane R. S., Strom S. C., Desai P. B. (2006). In vitro methods in human drug biotransformation research: implications for cancer chemotherapy. *Toxicology in Vitro*.

[13] Wu X., Wang J., Tan L. (2012). *In Vitro* ADME profiling using high-throughput rapidfire mass spectrometry: cytochrome P450 inhibition and metabolic stability assays. *Journal of Biomolecular Screening*.

[14] Trubetskoy O. V., Gibson J. R., Marks B. D. (2005). Highly miniaturized formats for in vitro drug metabolism assays using vivid fluorescent substrates and recombinant human cytochrome P450 enzymes. *Journal of Biomolecular Screening*.

[15] Hewitt N. J., Lechón M. J. G., Houston J. B. (2007). Primary hepatocytes: current understanding of the regulation of metabolic enzymes and transporter proteins, and pharmaceutical practice for the use of hepatocytes in metabolism, enzyme induction, transporter, clearance, and hepatotoxicity studies. *Drug Metabolism Reviews*.

[16] Chao P., Uss A. S., Cheng K. (2010). Use of intrinsic clearance for prediction of human hepatic clearance. *Expert Opinion on Drug Metabolism and Toxicology*.

[17] Chiba M., Ishii Y., Sugiyama Y. (2009). Prediction of hepatic clearance in human from *in vitro* data for successful drug development. *The AAPS Journal*.

[18] Niro R., Byers J. P., Fournier R. L., Bachmann K. (2003). Application of a convective-dispersion model to predict in vivo hepatic clearance from in vitro measurements utilizing cryopreserved human hepatocytes. *Current Drug Metabolism*.

[19] Naritomi Y., Terashita S., Kagayama A., Sugiyama Y. (2003). Utility of hepatocytes in predicting drug metabolism: comparison of hepatic intrinsic clearance in rats and humans *in vivo* and *in vitro*. *Drug Metabolism & Disposition*.

[20] Stringer R. A., Strain-Damerell C., Nicklin P., Houston J. B. (2009). Evaluation of recombinant cytochrome p450 enzymes as an in vitro system for metabolic clearance predictions. *Drug Metabolism and Disposition*.

[21] Chen Y., Liu L., Nguyen K., Fretland A. J. (2011). Utility of intersystem extrapolation factors in early reaction phenotyping and the quantitative extrapolation of human liver microsomal intrinsic clearance using recombinant cytochromes P450. *Drug Metabolism and Disposition*.

[22] Rowland M., Peck C., Tucker G. (2011). Physiologically-based pharmacokinetics in drug development and regulatory science. *Annual Review of Pharmacology and Toxicology*.

[23] Gerlowski L. E., Jain R. K. (1983). Physiologically based pharmacokinetic modeling: principles and applications. *Journal of Pharmaceutical Sciences*.

[24] Rostami-Hodjegan A. (2012). Physiologically based pharmacokinetics joined with in vitro-in vivo extrapolation of ADME: a marriage under the arch of systems pharmacology. *Clinical Pharmacology and Therapeutics*.

[25] Rostami-Hodjegan A., Tucker G. T. (2007). Simulation and prediction of in vivo drug metabolism in human populations from in vitro data. *Nature Reviews Drug Discovery*.

[26] Yap C. W., Xue Y., Li H. (2006). Prediction of compounds with specific pharmacodynamic, pharmacokinetic or toxicological property by statistical learning methods. *Mini-Reviews in Medicinal Chemistry*.

[27] Demir-Kavuk O., Bentzien J., Muegge I., Knapp E.-W. (2011). DemQSAR: predicting human volume of distribution and clearance of drugs. *Journal of Computer-Aided Molecular Design*.

[28] Simcyp http://www.simcyp.com/.

[29] Gastroplus http://www.simulations-plus.com/.

[30] Pelkonen O., Turpeinen M., Uusitalo J., Rautio A., Raunio H. (2005). Prediction of drug metabolism and interactions on the basis of in vitro investigations. *Basic and Clinical Pharmacology and Toxicology*.

[31] Buchan N. S., Rajpal D. K., Webster Y. (2011). The role of translational bioinformatics in drug discovery. *Drug Discovery Today*.

[32] Obach R. S., Lombardo F., Waters N. J. (2008). Trend analysis of a database of intravenous pharmacokinetic parameters in humans for 670 drug compounds. *Drug Metabolism & Disposition*.

[33] Mallo C., Zaidan R., Galy G. (1990). Pharmacokinetics of melatonin in man after intravenous infusion and bolus injection. *European Journal of Clinical Pharmacology*.

[34] Lei H. P., Yu X. Y., Xie H. T. (2010). Effect of St. John's wort supplementation on the pharmacokinetics of bupropion in healthy male Chinese volunteers. *Xenobiotica*.

[35] Hill S., Sikand H., Lee J. (2007). A case report of seizure induced by bupropion nasal insufflation. *Primary Care Companion to the Journal of Clinical Psychiatry*.

[36] Gengiah T. N., Holford N. H. G., Botha J. H., Gray A. L., Naidoo K., Karim S. S. A. (2012). The influence of tuberculosis treatment on efavirenz clearance in patients co-infected with HIV and tuberculosis. *European Journal of Clinical Pharmacology*.

[37] Chiappetta D. A., Hocht C., Taira C., Sosnik A. (2011). Oral pharmacokinetics of the anti-HIV efavirenz encapsulated within polymeric micelles. *Biomaterials*.

[38] Hayes M. J., Langman M. J. S., Short A. H. (1975). Changes in drug metabolism with increasing age: 2. Phenytoin clearance and protein binding. *British Journal of Clinical Pharmacology*.

[39] Drug Bank http://www.drugbank.ca.

[40] Paulson S. K., Vaughn M. B., Jessen S. M. (2001). Pharmacokinetics of celecoxib after oral administration in dogs and humans: effect of food and site of absorption. *Journal of Pharmacology and Experimental Therapeutics*.

[41] Moghadamnia A. A., Rostami-Hodjegan A., Abdul-Manap R., Wright C. E., Morice A. H., Tucker G. T. (2003). Physiologically based modelling of inhibition of metabolism and assessment of the relative potency of drug and metabolite: dextromethorphan vs. dextrorphan using quinidine inhibition. *British Journal of Clinical Pharmacology*.

[42] KuKanich B., Papich M. G. (2004). Plasma profile and pharmacokinetics of dextromethorphan after intravenous and oral administration in healthy dogs. *Journal of Veterinary Pharmacology and Therapeutics*.

[43] Brynne N., Stahl M. M., Hallen B. (1997). Pharmacokinetics and pharmacodynamics of tolterodine in man: a new drug for the treatment of urinary bladder overactivity. *International Journal of Clinical Pharmacology and Therapeutics*.

[44] Lefebvre R. A., Van Peer A., Woestenborghs R. (1997). Influence of itraconazole on the pharmacokinetics and electrocardiographic effects of astemizole. *British Journal of Clinical Pharmacology*.

[45] Lowry J. A., Kearns G. L., Abdel-Rahman S. M. (2003). Cisapride: a potential model substrate to assess cytochrome P4503A4 activity in vivo. *Clinical Pharmacology & Therapeutics*.

[46] Abel S., Russell D., Whitlock L. A., Ridgway C. E., Nedderman A. N., Walker D. K. (2008). Assessment of the absorption, metabolism and absolute bioavailability of maraviroc in healthy male subjects. *British Journal of Clinical Pharmacology*.

[47] Desta Z., Kerbusch T., Flockhart D. A. (1999). Effect of clarithromycin on the pharmacokinetics and pharmacodynamics of pimozide in healthy poor and extensive metabolizers of cytochrome P450 2D6 (CYP2D6). *Clinical Pharmacology & Therapeutics*.

[48] Brattstram C., Salve J., Jansson B. (2000). Pharmacokinetics and safety of single oral doses of sirolimus (rapamycin) in healthy male volunteers. *Therapeutic Drug Monitoring*.

[49] Wu H.-Y., Karnik S., Subhadarshini A. (2013). An integrated pharmacokinetics ontology and corpus for text mining. *BMC Bioinformatics*.

[50] FDA (2012). *Drug Interaction Studies-Study Design, Data Analysis, Implications for Dosing, and Labeling Recommendations*.

[51] Jamei M., Marciniak S., Feng K., Barnett A., Tucker G., Rostami-Hodjegan A. (2009). The Simcyp population-based ADME simulator. *Expert Opinion on Drug Metabolism and Toxicology*.

[52] Wattanachai N., Tassaneeyakul W., Rowland A. (2012). Effect of albumin on human liver microsomal and recombinant CYP1A2 activities: impact on in vitro-in vivo extrapolation of drug clearance. *Drug Metabolism and Disposition*.

[53] T’jollyn H., Snoeys J., Colin P. (2015). Physiology-based IVIVE predictions of tramadol from *in vitro* metabolism data. *Pharmaceutical Research*.

[54] Miners J. O., MacKenzie P. I., Knights K. M. (2010). The prediction of drug-glucuronidation parameters in humans: UDP-glucuronosyltransferase enzyme-selective substrate and inhibitor probes for reaction phenotyping and in vitroin vivo extrapolation of drug clearance and drug-drug interaction potential. *Drug Metabolism Reviews*.

